# Early respiratory morbidity in a multicultural birth cohort: the Generation R Study

**DOI:** 10.1007/s10654-012-9675-9

**Published:** 2012-04-04

**Authors:** Carmelo Gabriele, Lindsay M. Silva, Lidia R. Arends, Hein Raat, Henriëtte A. Moll, Albert Hofman, Vincent W. Jaddoe, Johan C. de Jongste

**Affiliations:** 1 The Generation R Study Group, Erasmus University Medical Centre, Sophia Children’s Hospital, Rotterdam, The Netherlands; 2 Department of Pediatrics, Erasmus University Medical Centre, Sophia Children’s Hospital, PO Box 2060, 3000 CB Rotterdam, The Netherlands; 3 Present Address: Department of Paediatrics, University Medical Centre, Utrecht, The Netherlands; 4 Department of Public Health, Erasmus University Medical Centre, Rotterdam, The Netherlands; 5 Institute of Psychology, Erasmus University, Rotterdam, The Netherlands; 6 Department of Biostatistics, Erasmus University Medical Centre, Rotterdam, The Netherlands; 7 Department of Epidemiology, Erasmus University Medical Centre, Rotterdam, The Netherlands

**Keywords:** Ethnicity, Infants, Pre and postnatal exposures, Prospective birth cohort, Respiratory symptoms

## Abstract

Ethnic disparities in the prevalence of asthma symptoms in children have been described. We evaluated to what extent the association between ethnic background and respiratory symptoms during the first 2 years of life could be explained by the mediating effect of risk factors for respiratory morbidity. The Generation R Study is a multiethnic, population-based birth cohort study. Pre and postnatal risk factors for respiratory morbidity were prospectively assessed by questionnaires. Information about ethnicity was available for 5,684 infants. The associations between ethnic background and lower respiratory symptoms at 12 and 24 months were evaluated with log-binomial regression models. Relative risks and 95 % confidence intervals (RR [95 % CI]) were computed for Cape Verdean, Moroccan, Antillean, Surinamese and Turkish ethnicity with Dutch ethnicity as the reference category. We found an increased risk of lower respiratory symptoms at 24 months in Antillean infants (1.32 [95 % CI 1.12–1.57]) that was mediated by early postnatal exposures (pets keeping, siblings, breastfeeding, daycare attendance, smoke exposure). Turkish infants also had an increased risk of lower respiratory symptoms at 12 and 24 months (1.14 [95 % CI 1.02–1.27] and 1.21 [95 % CI 1.07–1.38], respectively), partly explained by previous morbidity (eczema, infections and upper respiratory symptoms). There were no differences for Cape Verdean, Moroccan or Surinamese, as compared to Dutch infants. Hence, ethnic background was associated with respiratory symptoms during the first 2 years of life and this association was largely explained by mediating effects of known pre and postnatal risk factors for respiratory morbidity.

## Introduction

The prevalence of asthma symptoms varies worldwide, with the highest rates in children of countries with a Western lifestyle [1]. Variation in the prevalence of respiratory symptoms and asthma has been shown among children with different ethnic background living in the same urban area [2–4]. Previous cross-sectional studies have shown that in the US some ethnic groups, such as African-Americans and Porto Ricans, are more prone to develop asthma compared to Caucasian and Mexican-Americans and that these differences are mostly independent of socioeconomic variables [5–8]. Studies on migrants in Europe showed that children of Turkish ethnicity had a lower prevalence of atopic diseases compared to their German and Swedish peers [3, 9], with a positive association between higher levels of cultural adaptation in Turkish migrants and the prevalence of atopic diseases [10]. Furthermore, studies from US, Europe and Australia have demonstrated that the risk of respiratory symptoms in migrants increases in case of immigration early in life and is positively associated with the duration of residence, suggesting that environmental rather than genetic factors determine the risk of atopy and asthma [9, 11, 12]. Most asthma begins early in life [13] and the main symptoms reported in preschool children are wheezing, cough and breathlessness. Infants with these symptoms have an increased risk of developing asthma when compared with infants without [14]. Until now, only one longitudinal study conducted in the Netherlands evaluated respiratory morbidity from early infancy in children of different ethnicity. Koopman et al. [15] prospectively assessed the prevalence of respiratory symptoms in the first 2 years of life in a cohort of children with different ethnic backgrounds. The authors showed that symptoms of the respiratory tract were reported more frequently in non-Dutch children as compared with Dutch. However, the groups of children with non-Dutch ethnicities were relatively small, and the findings could be largely explained by differences in socioeconomic status [15]. Within the framework of a large prospective multiethnic birth cohort, we now examined the associations between ethnic background and symptoms of the lower respiratory tract in the first 2 years of life. We also assessed whether these associations could be explained by known risk factors for respiratory morbidity.

## Methods

### Study design

‘Generation R’ is a prenatally recruited population-based multicultural birth cohort in which 9,778 pregnant women and their children were enrolled in the city of Rotterdam (the Netherlands) between April 2002 and January 2006 [16, 17]. Pre and postnatal risk factors for respiratory morbidity were prospectively assessed by questionnaires administered in early (<18 weeks), mid (18–25 weeks) and late (>25 weeks) pregnancy and when the child was 6, 12 and 24 months of age. If needed, ethnic minorities were approached in their own language. Of the 9,778 women enrolled, 8,880 were included prenatally, 6,969 agreed to participate to the postnatal phase and 6,492 gave full consent to the use of postnatal data. Twin pregnancies (n = 50) were excluded and if the mother participated with more than one pregnancy (n = 488), only the first pregnancy was included in the analyses to avoid clustering (n = 5,954). As ethnic background was the main determinant of the study, infants with missing data on ethnicity (n = 270) were excluded, leaving 5,684 infants for the analyses. The Medical Ethical Committee of the Erasmus Medical Center, Rotterdam, approved the study and parents gave written informed consent.

### Definition of ethnicity

According to the Dutch standard classification [18], a child was considered to be of Dutch ethnicity if both parents were born in the Netherlands and from migrant origin if one parent was born abroad. If both parents were born abroad, mother’s country of origin was leading. A distinction was made among the non-Dutch minority populations in this study: Capeverdean, Moroccan, Dutch Antillean, Surinamese and Turkish, which represent the largest immigrant populations in the Netherlands, and ‘others’ (European, North American, Oceanean, Japanese, Indonesian, African, Asian, South and Central American).

### Outcomes

Wheezing, breathlessness, dry cough without a cold, persistent phlegm (having had phlegm on at least 4 days per week over a period of at least 3 months) and doctor-diagnosed asthma in the past year were assessed at 12 and 24 months by means of postal questionnaires. Questions on wheezing, breathlessness and cough were adapted from the ISAAC core questionnaires [19] and the question on persistent phlegm was based on the American Thoracic Society questionnaire for respiratory symptoms in childhood [20]. The combined variables lower respiratory symptoms (LRS) at 0–12 months and LRS at 12–24 months were considered positive if at least one of the above symptoms was reported at 12 or at 24 months, respectively.

### Mediators and confounders

The following risk factors for LRS, considered as intermediate in possible causal pathways between ethnicity and LRS [21], are referred to as potential mediators:

- Socioeconomic status (SES) defined by net monthly income of the household (<900, 900–2,200 or >2,200 Euro), maternal education (low, intermediate or high vocational training) and marital status (married/living together or living alone).
- Prenatal environment and perinatal characteristics. Prenatal smoke exposure by the mother and parental atopy were assessed by questionnaire; birth weight (kg) and gestational age (weeks) were obtained from hospital registries.
- Postnatal environment and nutrition. Based on the answer to pre-and postnatal questionnaires, the variable ‘pets keeping’ was grouped in 4 mutually exclusive categories (never exposed, prenatal exposure only, postnatal exposure only or pre and postnatal exposure). Breastfeeding and the number of siblings in the household were recorded at 6 months. Daycare attendance in the past 12 months was investigated at 12 and at 24 months. Postnatal environmental tobacco smoke exposure was assessed at 24 months.
- Previous morbidity. At 12 and 24 months doctor-diagnosed child allergy, upper respiratory symptoms (URS), eczema, respiratory and non-respiratory tract infections were assessed.
- Gender and age of the child at the time of completion of the 12 and 24 months’ questionnaires were included in all regression models as confounders.

### Statistical analysis

**Fig. 1:**
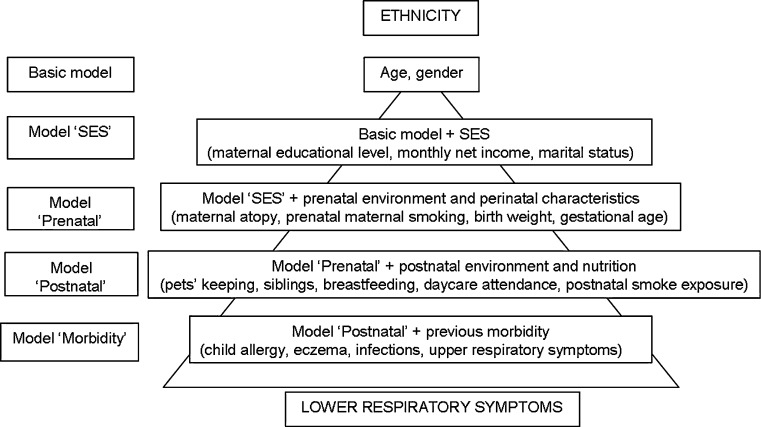
Hierarchical framework: variables near to the top influence those below

Categorical variables were compared with Chi-square tests. Continuous variables were not normally distributed and were compared with Mann–Whitney U-test or Kruskal–Wallis test, as appropriate. Multiple imputation was used to impute missing values of the outcomes, mediators and confounders [22, 23]. Using the PROC MI procedure in SAS 9.1.3, five imputed data sets were created, in which imputations were based on the relationships between all the variables included in this study. Estimated associations in each of the imputed datasets differed because of the variation introduced in the imputation of the missing values. These were averaged to give overall estimated associations. The associations between ethnic background and LRS during the first (0–12 months) or the second year (12–24 months) were evaluated in two separate log-binomial regression models adjusting for age and gender (basic model). Variables used in both models included: maternal education, net income, marital status, pre and postnatal smoke exposure, maternal atopy, birth weight, gestational age, pets keeping, breastfeeding, siblings and child allergy. The model fitted on LRS at 0–12 months included also daycare attendance, URS and infections at 0–12 months, whereas the model fitted on LRS at 12–24 months included daycare attendance, URS, infections at 12–24 months and LRS at 0–12 months. Each potential mediator was separately added to the basic model. Variables that individually caused a change of ≥10 % in the relative risk (RR) of any ethnic group compared to the Dutch in the basic model were included in subsequent regression models, taking into account the hierarchical relationship between the investigated mediating factors (Fig. 1) [24].

Variables associated with a >10 % change in RR of LRS at 0–12 months for any ethnic group were maternal education, net income, marital status, prenatal smoke exposure, birth weight, gestational age, pets keeping, breastfeeding, daycare attendance, eczema, respiratory and non-respiratory infections and URS. Variables associated with a change >10 % in RR of LRS at 12–24 months for any ethnic group included maternal education, net income, marital status, prenatal smoke exposure, birth weight, gestational age, pets keeping, siblings, breastfeeding, daycare attendance, postnatal smoke exposure, eczema, respiratory and non-respiratory infections and URS. Results of the log-binomial regression models are reported as RR and 95 % confidence intervals [95 % CI]. Statistical analyses were performed using the Statistical Package of Social Sciences version 15 for Windows (SPSS Inc, Chicago, IL, USA) and the Statistical Analysis System (SAS) for Windows, version 9.1.3.

## Results

### Study population

The ethnic background of the study population was Dutch in 56.6 %, Cape Verdean in 2.9 %, Moroccan in 6.2 %, Antillean in 3.1 %, Surinamese in 7 %, Turkish in 7.6 % and ‘others’ in 16.6 %. As the group ‘others’ represented a heterogeneous population of infants, the results were reported, but could not be meaningfully interpreted. The general characteristics of the different ethnic groups are presented in Table 1 (imputed data).

**Table 1:** General characteristics of the study population (imputed data)

	Dutch (N = 3,217)	Cape Verdean (N = 163)	Moroccan (N = 350)	Antillean (N = 178)	Surinamese (N = 400)	Turkish (N = 434)	Others (N = 942)
Male gender	50 %	49 %	49 %	60 %^†^	46 %	47 %	51 %
Educational level mother*							
High	59 %	13 %	14 %	19 %	19 %	14 %	56 %
Intermediate	38 %	63 %	59 %	67 %	68 %	51 %	34 %
Low	3 %	24 %	27 %	14 %	13 %	35 %	10 %
Monthly family net income*							
>2,200 Euro	75 %	11 %	11 %	20 %	33 %	14 %	53 %
900–2,200 Euro	21 %	51 %	68 %	40 %	45 %	62 %	35 %
<900 Euro	4 %	38 %	21 %	40 %	22 %	23 %	12 %
Mother married/living together	92 %	58 %^‡^	91 %	46 %^‡^	66 %^‡^	95 %	85 %^‡^
Prenatal maternal smoking	24 %	22 %	7 %^‡^	31 %^†^	29 %^†^	35 %^‡^	20 %^‡^
Maternal atopy	39 %	35 %	37 %	46 %	36 %	33 %^†^	35 %^†^
Birth weight: mean (SD) g	3.485 (0.5)^§^	3.303 (0.5)	3.523 (0.5)	3.174 (0.6)	3.184 (0.6)	3.387 (0.5)	3.383 (0.5)
Gestational age: mean (SD) weeks	40.0 (1.7)^§^	39.9 (1.4)	40.3 (1.5)	39.5 (1.8)	39.5 (1.8)	39.9 (1.8)	39.9 (1.7)
Pets keeping*	55 %	80 %	92 %	77 %	75 %	85 %	72 %
Never	7 %	8 %	4 %	7 %	9 %	7 %	5 %
Only prenatal	6 %	7 %	1 %	4 %	5 %	3 %	4 %
Only postnatal	32 %	5 %	3 %	12 %	11 %	5 %	19 %
Pre and postnatal							
Breastfeeding	28 %	19 %^†^	27 %	15 %^‡^	19 %^‡^	40 %^‡^	39 %^‡^
Siblings	34 %	50 %^‡^	57 %^‡^	34 %	40 %^†^	51 %^‡^	33 %
Daycare attendance at 0–12 months	92 %	87 %	66 %^‡^	79 %^‡^	85 %^‡^	59 %^‡^	82 %^‡^
Daycare attendance at 12–24 months	89 %	88 %	51 %^‡^	82 %^†^	81 %^‡^	48 %^‡^	78 %^‡^
Postnatal smoke exposure	30 %	25 %	24 %	40 %^†^	42 %^‡^	56 %^‡^	30 %
Child allergy	6 %	6 %	7 %	8 %	6 %	3 %	7 %
URS at 0–12 months	38 %	50 %	46 %^†^	40 %	50 %^‡^	44 %	43 %^†^
URS at 12–24 months	30 %	39 %	31 %	36 %	35 %	37 %^†^	28 %
Eczema at 0–12 months	31 %	34 %	48 %^‡^	37 %	48 %^‡^	36 %	35 %
Eczema at 12–24 months	22 %	27 %	16 %	30 %	31 %^‡^	14 %^‡^	22 %
Infections at 0–12 months							
No	18 %	23 %	20 %	19 %	20 %	14 %	20 %
URTI or LRTI	68 %	63 %	76 %	76 %	70 %	84 %	68 %
Other infections	14 %	14 %	4 %	5 %	10 %	2 %	12 %
Infections at 12–24 months*							
No	10 %	5 %	11 %	6 %	11 %	5 %	10 %
URTI or LRTI	71 %	86 %	78 %	85 %	78 %	89 %	75 %
Other infections	19 %	9 %	11 %	9 %	11 %	6 %	15 %

*SD* standard deviation, *URS* upper respiratory symptoms, *URTI* upper respiratory tract infections, *LRTI* lower respiratory tract infections

^†^ *p* < 0.05 compared to Dutch (Chi-square test)

^‡^ *p* < 0.01 compared to Dutch (Chi-square test)

* *p* < 0.01, Chi-square test

^§^ *p* < 0.01, Kruskal–Wallis test

Risk factors for lower respiratory symptoms at 12 months were male gender, allergy, daycare attendance, maternal atopy, high maternal educational level, high monthly net income, respiratory tract infections, URS, eczema, no breastfeeding at 6 months. At 24 months, risk factors for LRS were male gender, allergy, daycare attendance, exposure to pre and postnatal tobacco smoke, maternal atopy, having a single mother, eczema, respiratory tract infections, URS, having no siblings, non-exposure to pre and postnatal pets, no breastfeeding at 6 months. The prevalence of respiratory symptoms in different ethnic groups showed that non-Dutch ethnic groups were more likely to have missing data on LRS both at 0–12 and at 12–24 months (Tables 2,3; not imputed data).

**Table 2:** Prevalence of respiratory symptoms at 0–12 months (not imputed data)

	Dutch (N = 3,217)	Cape Verdean (N = 163)	Moroccan (N = 350)	Antillean (N = 178)	Surinamese (N = 400)	Turkish (N = 434)	Others (N = 942)
Wheezing							
No	59 %	30 %	35 %	39 %	4 %3	40 %	54 %
Yes	26 %	17 %	10 %	18 %	15 %	20 %	17 %
Missing	15 %	53 %	55 %	43 %	41 %	40 %	29 %
Breathlessness							
No	64 %	41 %	37 %	45 %	49 %	50 %	59 %
Yes	21 %	7 %	8 %	12 %	9 %	10 %	12 %
Missing	15 %	52 %	55 %	43 %	42 %	40 %	29 %
Dry cough without a cold							
No	64 %	39 %	36 %	44 %	47 %	46 %	55 %
Yes	20 %	8 %	10 %	11 %	11 %	13 %	15 %
Missing	16 %	53 %	54 %	45 %	42 %	41 %	30 %
Persistent phlegm							
No	75 %	36 %	36 %	41 %	49 %	44 %	59 %
Yes	9 %	10 %	10 %	15 %	10 %	14 %	10 %
Missing	16 %	54 %	54 %	44 %	41 %	14 %	31 %
Doctor-diagnosed asthma							
No	82 %	45 %	43 %	53 %	56 %	56 %	67 %
Yes	2 %	1 %	2 %	4 %	2 %	2 %	3 %
Missing	16 %	53 %	55 %	43 %	42 %	42 %	30 %
LRS at 0–12 months							
No	41 %	23 %	28 %	24 %	31 %	26 %	40 %
Yes	45 %	25 %	18 %	33 %	28 %	34 %	31 %
Missing	14 %	52 %	54 %	43 %	41 %	40 %	29 %

**Table 3:** Prevalence (%) of respiratory symptoms at 12–24 months (not imputed data)

	Dutch (N = 3,217)	Cape Verdean (N = 163)	Moroccan (N = 350)	Antillean (N = 178)	Surinamese (N = 400)	Turkish (N = 434)	Others (N = 942)
Wheezing							
No	68 %	40 %	41 %	34 %	45 %	46 %	59 %
Yes	16 %	12 %	7 %	14 %	14 %	14 %	13 %
Missing	16 %	48 %	52 %	52 %	41 %	40 %	28 %
Breathlessness							
No	68 %	43 %	43 %	36 %	52 %	53 %	62 %
Yes	16 %	9 %	6 %	12 %	7 %	7 %	10 %
Missing	16 %	48 %	51 %	52 %	41 %	40 %	28 %
Dry cough without a cold							
No	64 %	37 %	40 %	34 %	47 %	43 %	56 %
Yes	20 %	13 %	9 %	15 %	13 %	20 %	16 %
Missing	16 %	50 %	51 %	51 %	40 %	37 %	28 %
Persistent phlegm							
No	77 %	38 %	45 %	39 %	50 %	51 %	65 %
Yes	7 %	14 %	3 %	10 %	9 %	12 %	7 %
Missing	16 %	48 %	51 %	51 %	41 %	37 %	28 %
Doctor-diagnosed asthma							
No	82 %	49 %	48 %	46 %	57 %	60 %	71 %
Yes	2 %	3 %	1 %	2 %	3 %	2 %	2 %
Missing	16 %	48 %	51 %	52 %	40 %	38 %	27 %
LRS at 12–24 months							
No	47 %	28 %	32 %	21 %	33 %	30 %	43 %
Yes	38 %	25 %	16 %	28 %	26 %	33 %	30 %
Missing	15 %	47 %	52 %	51 %	41 %	37 %	27 %

### Ethnicity and lower respiratory symptoms

**Fig. 2:**
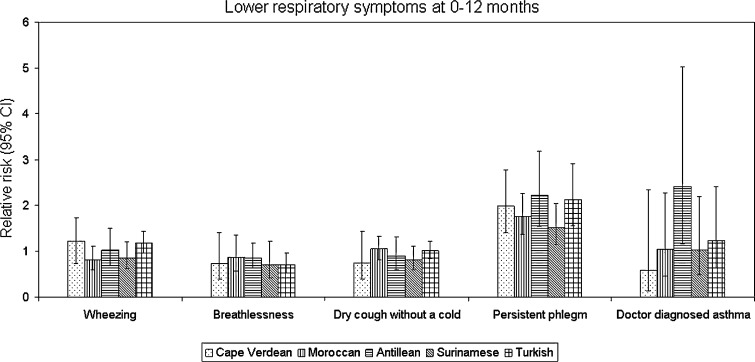
Relative risk (RR) of respiratory symptoms at 0–12 months for each ethnic group compared to Dutch. *Bars* represent 95 % CI

**Fig. 3:**
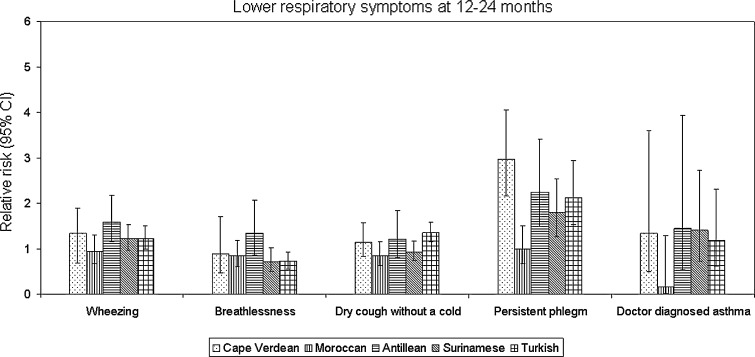
Relative risk (RR) of respiratory symptoms at 12–24 months for each ethnic group compared to Dutch. *Bars* represent 95 % CI

The log-binomial regression models adjusted for gender and age showed that Turkish children had a reduced relative risk of breathlessness at 12 and 24 months and an increased risk of dry cough without a cold at 24 months, whereas Antilleans were more likely to have doctor-diagnosed asthma at 12 months and wheezing at 24 months, as compared to Dutch. Also, all non-Dutch ethnic groups had increased risk of persistent phlegm at 12 months and all except Moroccans also at 24 months (Figs. 2,3).

The basic model fitted on LRS at 0–12 months (Table 4) showed that infants with Turkish background had increased risk of LRS compared to their Dutch peers (RR [95 % CI] 1.14 [1.02–1.27]). Following the hierarchical levels, socioeconomic factors were added to the basic model (model ‘SES’) and this caused a 35 % increase of the RR of Turkish vs. Dutch (RR 1.19 [1.06–1.34]). Adding prenatal exposures and birth data had no consistent effect on the RR. The model that included also nutrition and postnatal exposures (model ‘Postnatal’) caused a further increase of the RR of LRS of Turkish vs. Dutch (RR 1.21 [1.06–2.37]), due to less daycare in Turkish infants, as daycare was a risk factor for LRS at 0–12 months (RR 1.92 [1.58–2.33]). Adding previous morbidity variables reduced the RR of LRS at 0–12 months of Turkish versus Dutch to 1.20 [1.07–1.35] as infections at 12 months, which were a risk factor for LRS, were more frequently reported by Turkish than by Dutch parents. The other ethnic groups did not show any different risk of LRS at 0–12 months compared to Dutch (Table 4).

**Table 4:** Hierarchical log-binomial regression models models fitted on LRS at 0–12 months (n = 5,684)

	Basic model	Model ‘SES’	Model ‘Prenatal’	Model ‘Postnatal’	Model ‘Morbidity’
Ethnicity					
Dutch	Reference	Reference	Reference	Reference	Reference
Cape Verdean	1.07 (0.88–1.32)	1.07 (0.88–1.32)	1.08 (0.88–1.33)	1.02 (0.85–1.25)	1.06 (0.87–1.29)
Moroccan	0.95 (0.81–1.12)	0.99 (0.84–1.18)	1.01 (0.85–1.19)	1.01 (0.87–1.18)	0.92 (0.80–1.07)
Antillean	1.12 (0.89–1.41)	1.11 (0.89–1.38)	1.10 (0.88–1.38)	1.09 (0.88–1.35)	1.09 (0.94–1.26)
Surinamese	0.95 (0.82–1.10)	0.96 (0.83–1.11)	0.96 (0.83–1.12)	0.95 (0.81–1.12)	0.91 (0.80–1.04)
Turkish	1.14 (1.02–1.27)	1.19 (1.06–1.34)	1.19 (1.06–1.33)	1.21 (1.06–1.37)	1.20 (1.07–1.35)
Others	0.89 (0.83–0.96)	0.89 (0.82–0.97)	0.90 (0.83–0.97)	0.92 (0.85–0.99)	0.92 (0.84–0.99)

Results are reported as RR (95 % CI)

Basic model: Ethnicity, age, gender. Model ‘SES’: Basic model + educational level of the mother, income, marital status. Model ‘Prenatal’: Model ‘SES’ + maternal smoking during pregnancy, birth weight, gestational age. Model ‘Postnatal’: Model ‘Prenatal’ + pets keeping, breastfeeding, daycare attendance at 1 year. Model ‘Morbidity’: Model ‘Postnatal’ + eczema at 1 year, infections at 1 year, upper respiratory symptoms at 1 year

The basic model fitted on LRS at 12–24 months showed that Antillean and Turkish children had an increased risk of LRS in the second year compared to Dutch (Table 5). The association between Moroccan, Cape Verdean or Surinamese ethnicity and LRS at 12–24 months was not significant in the basic model. Adding socioeconomic factors (model ‘SES’) caused a 34.4 % reduction of the RR for Antillean to 1.21 [1.02–1.43], as Antillean mothers were more likely unmarried or living alone, this being a risk factor for LRS at 12–24 months. Prenatal exposures and birth data did not change the RR across ethnic groups. The model ‘Postnatal’ included nutrition and postnatal exposures, which mediated large part of the increased risk of LRS in Antillean at 12–24 months (1.16 [0.96–1.40]). Previous morbidity mediated part of the increased risk of LRS in Turkish and Antillean children as adding this set of variables reduced the RR across all ethnic groups (model ‘Morbidity’). The association between Turkish ethnicity and increased risk of LRS did not reach statistical significance in the second year (RR 1.06 [0.96–1.17]). The mediating effect of previous morbidity was related to the higher prevalence of risk factors for LRS at 12–24 months in Turkish compared to Dutch infants.

**Table 5:** Hierarchical log-binomial regression models models fitted on LRS at 12–24 months (n = 5,684)

	Basic model	Model ‘SES’	Model ‘Prenatal’	Model ‘Postnatal’	Model ‘Morbidity’
Ethnicity					
Dutch	Reference	Reference	Reference	Reference	Reference
Cape Verdean	1.18 (0.92–1.51)	1.09 (0.84–1.42)	1.11 (0.85–1.45)	1.05 (0.80–1.39)	0.95 (0.79–1.16)
Moroccan	0.92 (0.75–1.12)	0.91 (0.74–1.12)	0.93 (0.76–1.15)	0.95 (0.78–1.15)	0.88 (0.73–1.06)
Antillean	1.32 (1.12–1.57)	1.21 (1.02–1.43)	1.21 (1.01–1.44)	1.16 (0.96–1.40)	1.07 (0.92–1.25)
Surinamese	1.04 (0.91–1.18)	0.99 (0.86–1.13)	0.99 (0.87–1.14)	0.97 (0.84–1.11)	0.97 (0.85–1.10)
Turkish	1.21 (1.07–1.38)	1.21 (1.05–1.40)	1.22 (1.05–1.41)	1.22 (1.05–1.42)	1.06 (0.96–1.17)
Others	0.94 (0.85–1.05)	0.93 (0.83–1.04)	0.94 (0.84–1.04)	0.94 (0.85–1.05)	0.98 (0.90–1.06)

Results are reported as RR (95 % CI)

Basic model: Ethnicity, age, gender. Model ‘SES’: Basic model + educational level of the mother, income, marital status. Model ‘Prenatal’: Model ‘SES’ + maternal smoking during pregnancy, birth weight, gestational age. Model ‘Postnatal’: Model ‘Prenatal’ + pets keeping, siblings, breastfeeding, daycare attendance at 2 years, postnatal smoke exposure. Model ‘Morbidity’: Model ‘Postnatal’ + eczema at 2 years, infections at 2 years, upper respiratory symptoms at 2 years and lower respiratory symptoms at 0–12 months

No difference was observed in the risk of LRS at 12–24 months between Dutch and Cape Verdean, Moroccan or Surinamese infants.

## Discussion

In a prospective birth cohort study we showed associations between ethnicity and the risk of lower respiratory symptoms. Compared to Dutch infants, Antillean infants had an increased risk of lower respiratory symptoms at 24 months, which was mediated by postnatal exposures. Infants of Turkish ethnicity reported more often infections, upper respiratory symptoms and eczema than Dutch in the first 2 years of life, which partly explained their increased risk of respiratory symptoms both at 12 and 24 months. The risk of lower respiratory symptoms in the first 2 years of life was not different in Cape Verdean, Moroccan or Surinamese infants, as compared to Dutch infants.

Our study focused on differences in respiratory morbidity between ethnic groups, coming from a multicultural urban society. The results are consistent with earlier findings of the PIAMA birth cohort, showing an increased risk of respiratory symptoms before the age of 2 years in Turkish infants living in the Netherlands [15]. However, the relatively small size of the non-Dutch groups in that study did not allow for a comparison between ethnic minorities and the differences observed disappeared after adjustment for socioeconomic indices [15]. In our study, ethnic groups had different prevalences of socioeconomic indicators of deprivation. Overall, ethnic minorities were more deprived than the indigenous Dutch population but in different ways, suggesting that different lifestyles and the behavioral adaptations to the environment of ethnic minorities may mask risk factors for respiratory symptoms during infancy.

It has been shown that daycare attendance is associated with frequent wheezing in the preschool years, but not afterwards [25]. In our study, daycare attendance suppressed part of the association between Turkish ethnicity and LRS at 12 and at 24 months, suggesting that if non-Dutch children had used more daycare, their risk of LRS would be greater than observed. However, the effect of daycare attendance on LRS is mediated by respiratory infections [26]; therefore, it is difficult to disentangle the different role played by daycare attendance and by infections. We found that both respiratory and non-respiratory infections were associated with increased risk of LRS in the first 2 years of life and, as patterns of respiratory diseases will become more clear, we will be able to assess whether the overall burden of infections may predispose towards a certain asthma phenotype.

Some possible limitations to our study have to be considered. We defined the ethnic background of infants according to the Dutch standard classification [18]. This classification is objective, reproducible and can be easily applied in epidemiological studies, allowing comparison with future studies. However, some misclassification might have occurred as third generation migrants were labelled Dutch and were hence not distinguished. This would reduce the contrast between Dutch and other ethnic groups, and hence the effect size of Turkish ethnicity on LRS.

In the current study we used standard respiratory questionnaires for schoolchildren, which have shown satisfactory repeatability but may not be optimal for infants and preschool children [27]. A recent study by Strippoli et al. [28], showed poorer repeatability in infants for questions regarding cough and upper respiratory symptoms compared to wheeze and shortness of breath. However, our study did not focus on wheezing alone, but the outcomes included the combined variables LRS at 0–12 and LRS at 12–24 months, which were considered positive if at least one of the investigated symptoms occurred. Also, a study by Michel et al. [29] conducted in the UK, showed that parents’ understanding of the term ‘wheeze’ was different between English speaking and non-English speaking parents. Yet, non-native Dutch in our study were approached in their own language and we did not found a systematic difference in reported respiratory symptoms between Dutch and non-Dutch parents, as shown by the higher risk of LRS in Turkish and the lower risk in Moroccans, as compared to Dutch. Therefore, we consider it unlikely that misclassification of the outcome variables occurred. We cannot exclude that the differences between the ethnic groups might be partly explained by cultural differences, different attitudes towards the use of the medical system and different cultural reporting of the symptoms.

A possible limitation of the current study is represented by the assessment of postnatal exposure to environmental tobacco smoke, which was only evaluated at 24 months and might have been susceptible of recall bias with regard to the analysis at 12 months. Yet, this might partly explain why a significant association between LRS and smoke exposure was found only at 12–24 months.

Missing data on the outcome variables were not completely at random, therefore a complete-case analysis was likely to introduce biased results [23] and would lead to loss of a large number of study subjects in the multivariate analyses. In order to overcome this issue, we imputed the outcome variables using the predictors under study, thus minimizing the possible bias [30].

Do our findings have practical implications? It would seem that reduction of the prevalence of infections and symptoms of the upper airways early in life could lead to a parallel reduction of the burden of lower respiratory symptoms in non-Dutch infants up to the levels of their Dutch peers. Environmental exposures could not entirely explain the association between ethnic background and respiratory symptoms, and it could be argued that ethnicity-specific genetic factors and gene-by-environment interactions among ethnic groups might predispose infants to the development of respiratory symptoms [31]. A longer follow up of our cohort will reveal whether the increased prevalence of respiratory symptoms in certain ethnic groups represents a temporary association with respiratory infections in early childhood, or predicts progression to chronic persistent symptoms, including asthma. Appropriate focus on prevention of environmental factors as identified might favorably influence such progression.

In conclusion, we found associations between ethnic background and respiratory symptoms in the first 2 years of life that could be largely explained by environmental exposures.

## Abbreviations

LRS: Lower respiratory symptoms
LRTI: Lower respiratory tract infections
RR: Relative risk
SES: Socio-economic status
URS: Upper respiratory symptoms
URTI: Upper respiratory tract infections
